# Supplementary material for: Dissection of multiple sclerosis genetics identifies B and CD4+ T cells as driver cell subsets
Source: Genome Biol. 2022 Jun 7;23:127. doi: 10.1186/s13059-022-02694-y (PMC9175345; doi:10.1186/s13059-022-02694-y)

**Additional File 1**

**Supplementary Figures**

**Figure S1:** Correlation (Pearson’s r^2^) in ATAC-seq profiles across hematopoietic cell types.


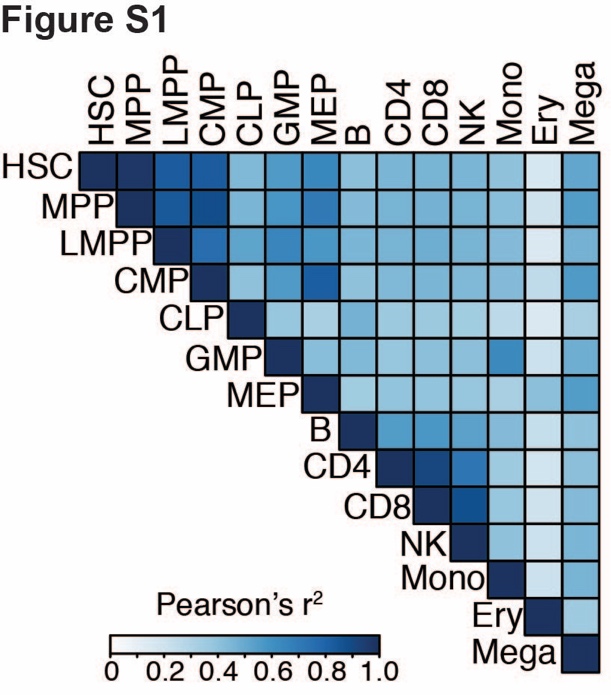


**Figure S2:** LDSC enrichments for MS GWAS in cell-type specific ATAC-seq peaks in each of the nine mature hematopoietic cell type. Y-axis shows –log_10_(p-value) of the LDSC heritability enrichment.


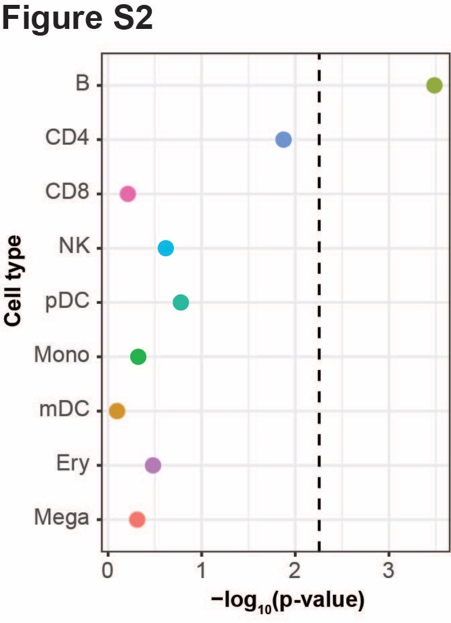


**Figure S3: A:** Enrichments of GWAS results from 10 neuropsychiatric or autoimmune conditions in OCRs across various hematopoietic cell types. **B:** LDSC coefficient p-values in the joint model across hematopoietic cell ATAC-seq in 10 neuropsychiatric or autoimmune conditions. For **A** and **B**, boxes are shaded by –log_10_(p-value), with darker shading reflecting more statistical significance, and statistically significant p-values (p-values<3.13x10^-3^) are starred.


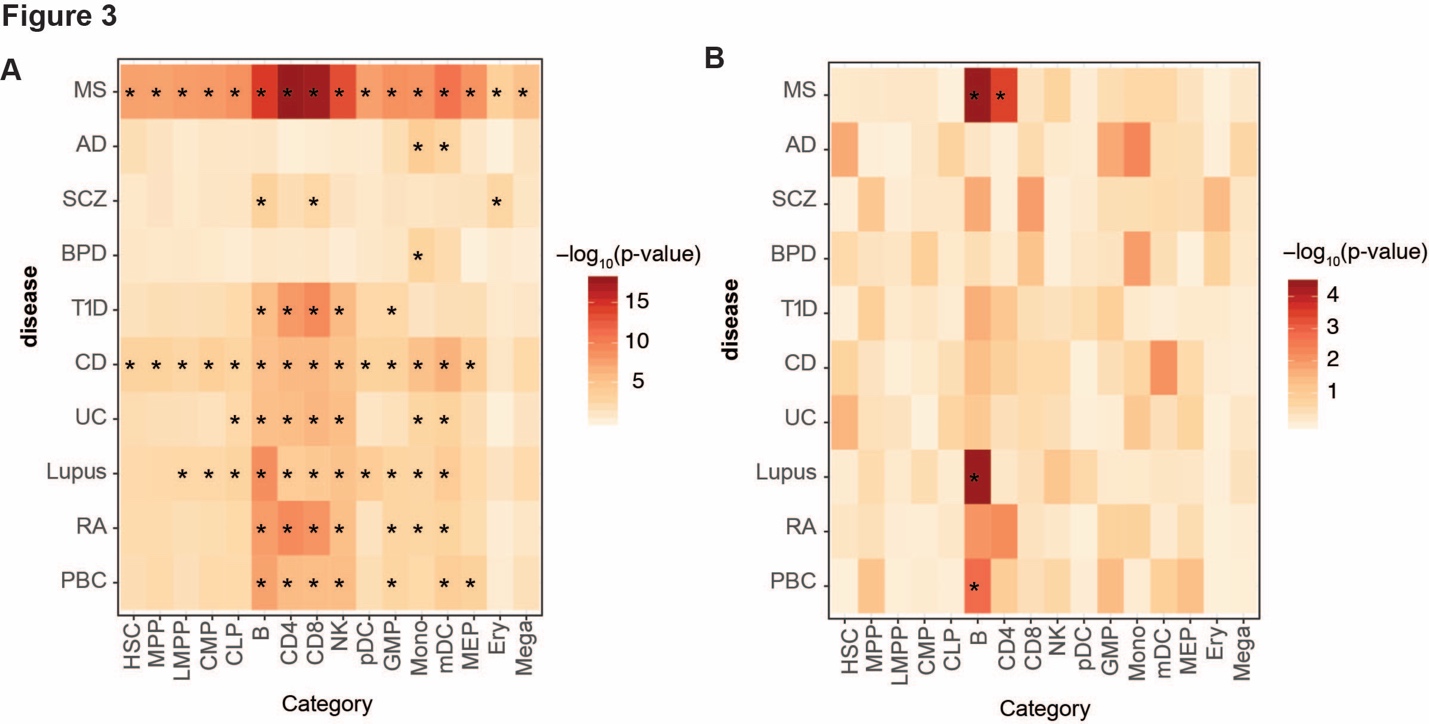


**Figure S4: A.** LDSC enrichment results for MS GWAS enrichment in T4cm OCRs from treated and MS treated patients in a joint model. **B.** Stratified LDSC enrichment results for MS GWAS enrichment in cMBc OCRs from treated and MS treated patients in a joint model. Heights of the circles reflect stratified LDSC coefficient p-values. Sizes of the circles are proportional to the enrichment p-values for that given cell type, with larger circles reflecting more significant p-values.

**
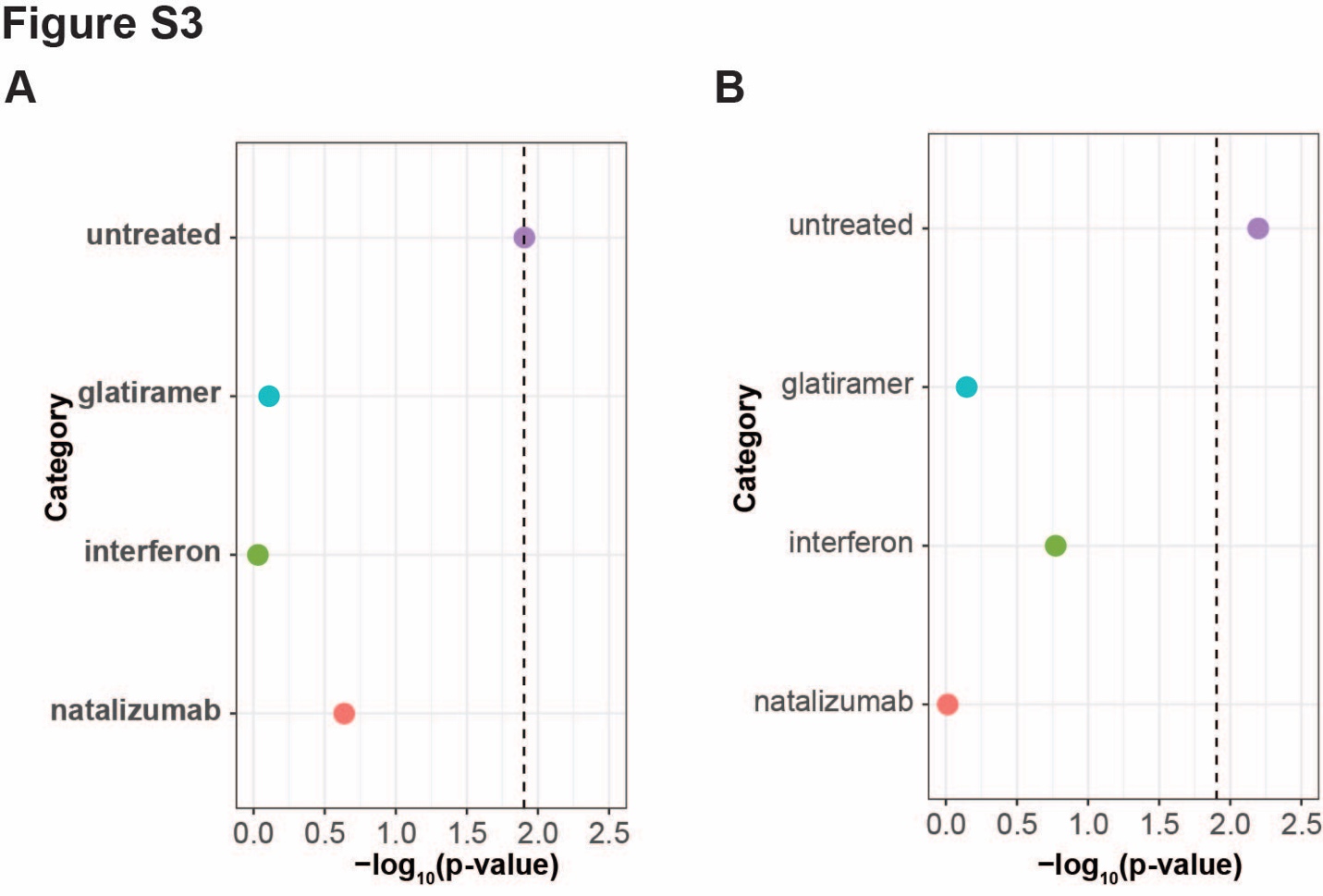
**

**Figure S5:** A: LDSC enrichment p-values for MS GWAS data in CD4^+^ T cell ChIP-seq peaks of various histone markers. Y-axis shown as –log_10_(p-value). **B:** Same as Figure S3A, except performed for B cell ChIP-seq histone markers.


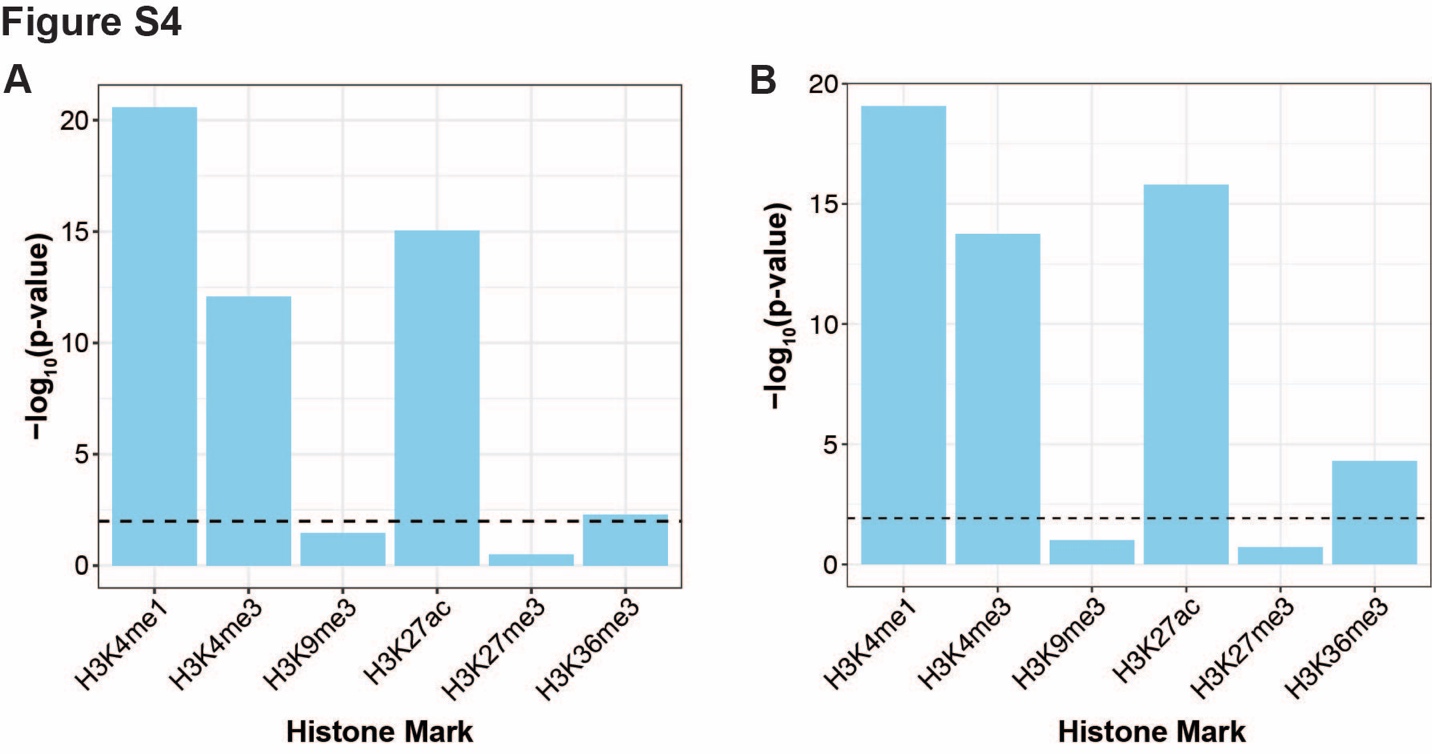


**Figure S6: A:** LDSC enrichment p-values for chromHMM chromatin states in T_h_17 CD4+ T cells from ENCODE. **B:** Same as Figure S5A, except for B cells from ENCODE. TssA: Active TSS; PromU: Promoter Upstream TSS; PromD1: Promoter Downstream TSS 1; PromD2: Promoter Downstream TSS 2; Tx5: Transcribed - 5' preferential; Tx: Strong transcription; Tx3: Transcribed - 3' preferential; TxWk: Weak transcription; TxReg: Transcribed & regulatory (Prom/Enh); TxEnh5: Transcribed 5' preferential and Enh; TxEnh3: Transcribed 3' preferential and Enh; TxEnhW: Transcribed and Weak Enhancer; EnhA1: Active Enhancer 1; EnhA2: Active Enhancer 2; EnhAF: Active Enhancer Flank; EnhW1: Weak Enhancer 1; EnhW2: Weak Enhancer 2; EnhAc: Primary H3K27ac possible Enhancer; DNase: Primary DNase; ZNF: ZNF genes & repeats; Het: Heterochromatin; PromP: Poised Promoter; PromBiv: Bivalent Promoter; ReprPC: Repressed Polycomb.


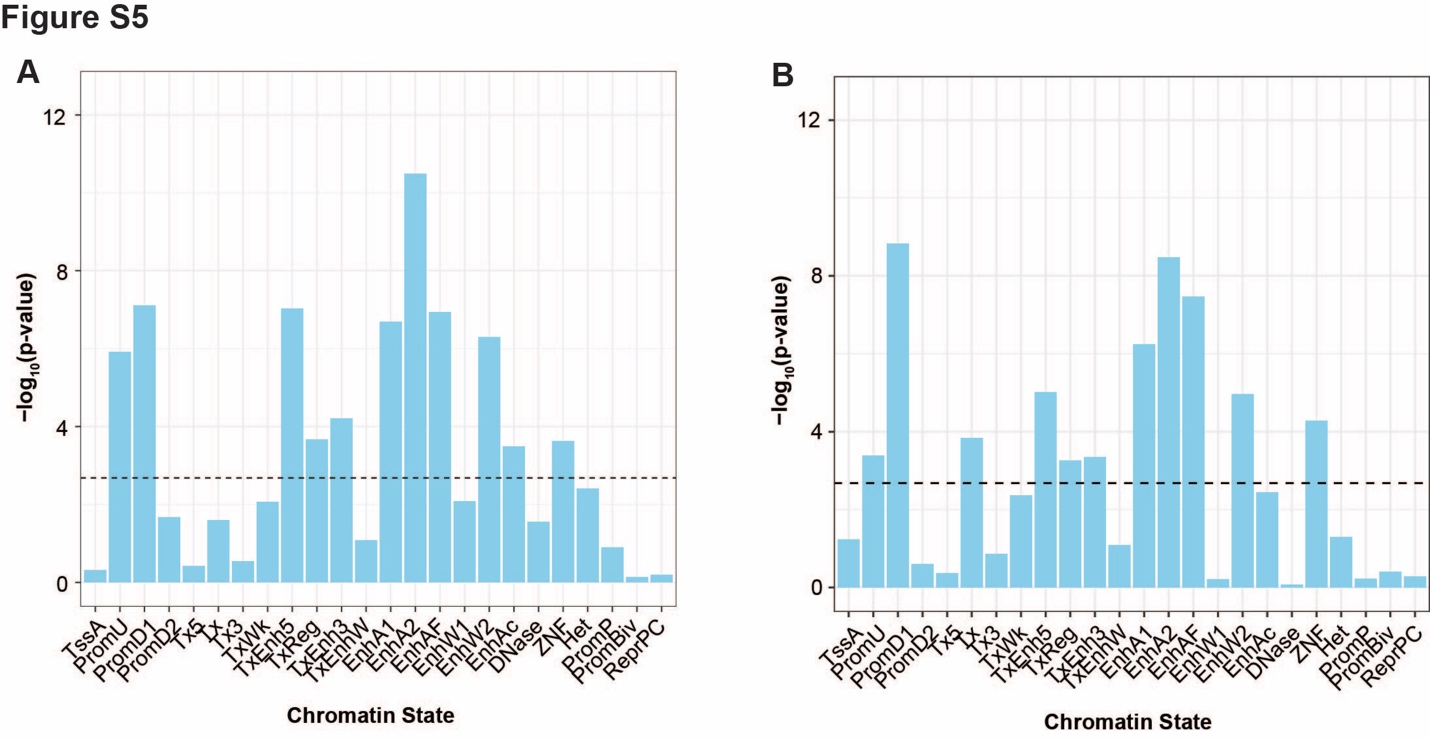


**Figure S7:** **A:** Histogram of the number of credible set (CS) variants across loci. **B:** Histogram of the PICS probability of the top variant in each locus.

**
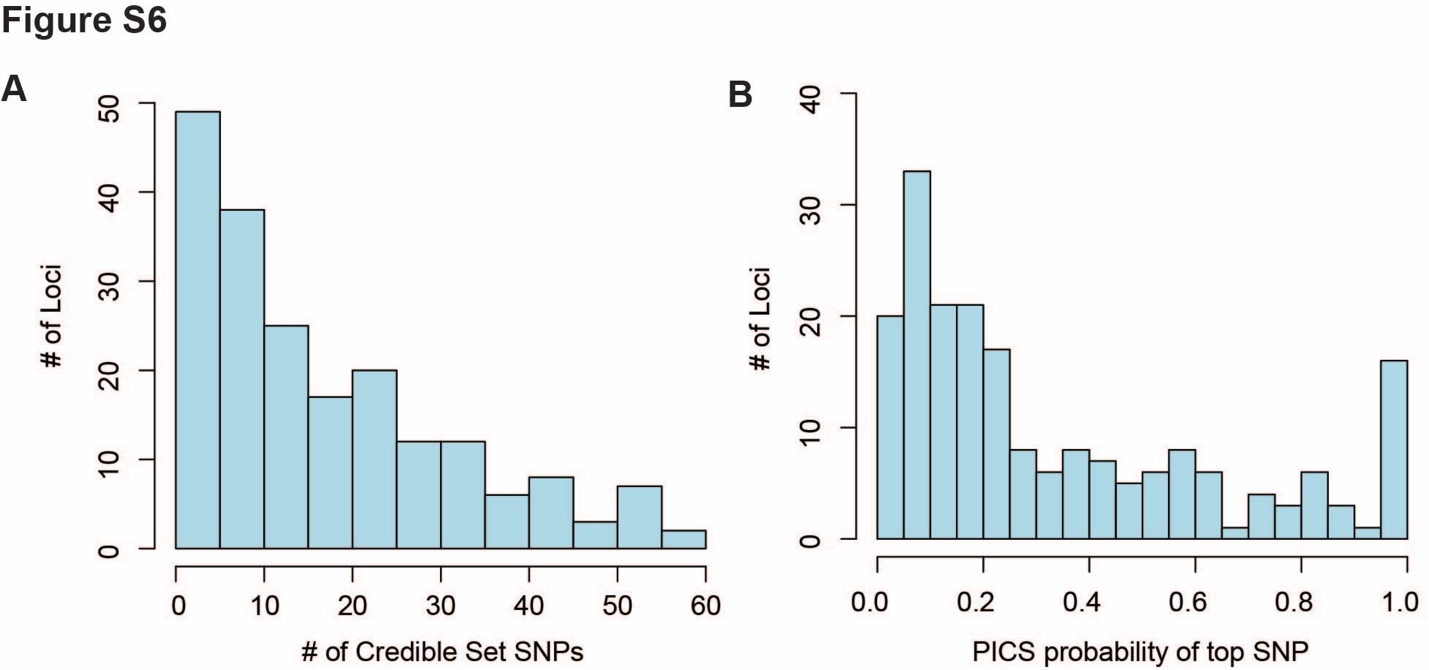
**

**Figure S8:** Number of MS GWAS loci (out of 200) with at least one CS SNP overlapping an ATAC-seq peak in the listed cell types.


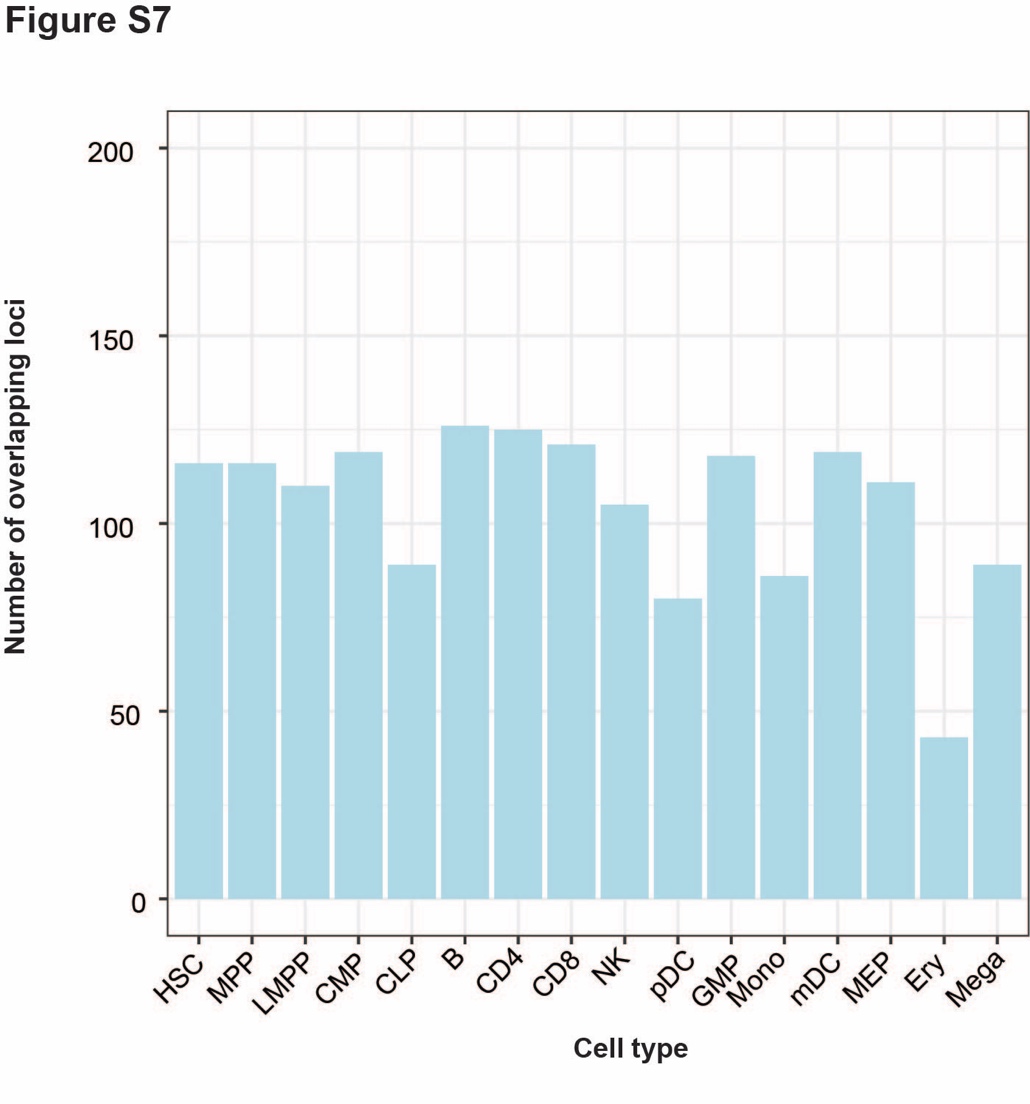


**Figure S9: Colocalization of MS GWAS loci with DICE CD4 T and B cell eQTLs.** Number of locus-gene pairs are depicted. The “Overlap” variable indicates locus-eGene pairs for which the MS locus overlapped with OCRs and PCHiC loops. THSTAR: Th1/17, TREG_NAIVE: naïve T regs, TREG_MEM: memory T regs, CD4_STIM: stimulated CD4 T, nB: naïve B cells.

**Figure S10: Colocalization enrichment of MS GWAS loci with DICE CD4 T and B cell eQTLs.** Per cell subtype OCR+loop enrichment was estimated with Fisher’s exact for the 2x2 table of a locus-eGene being colocalized vs. overlapping OCR+loop within either CD4 T cells or B cells. The dashed line depicts threshold for Bonferroni correction. THSTAR: Th1/17, TREG_NAIVE: naïve T regs, TREG_MEM: memory T regs, CD4_STIM: stimulated CD4 T, nB: naïve B cells.

**Figure S11:** Protein-protein interaction communities of putative causal genes in CD4 T cells.


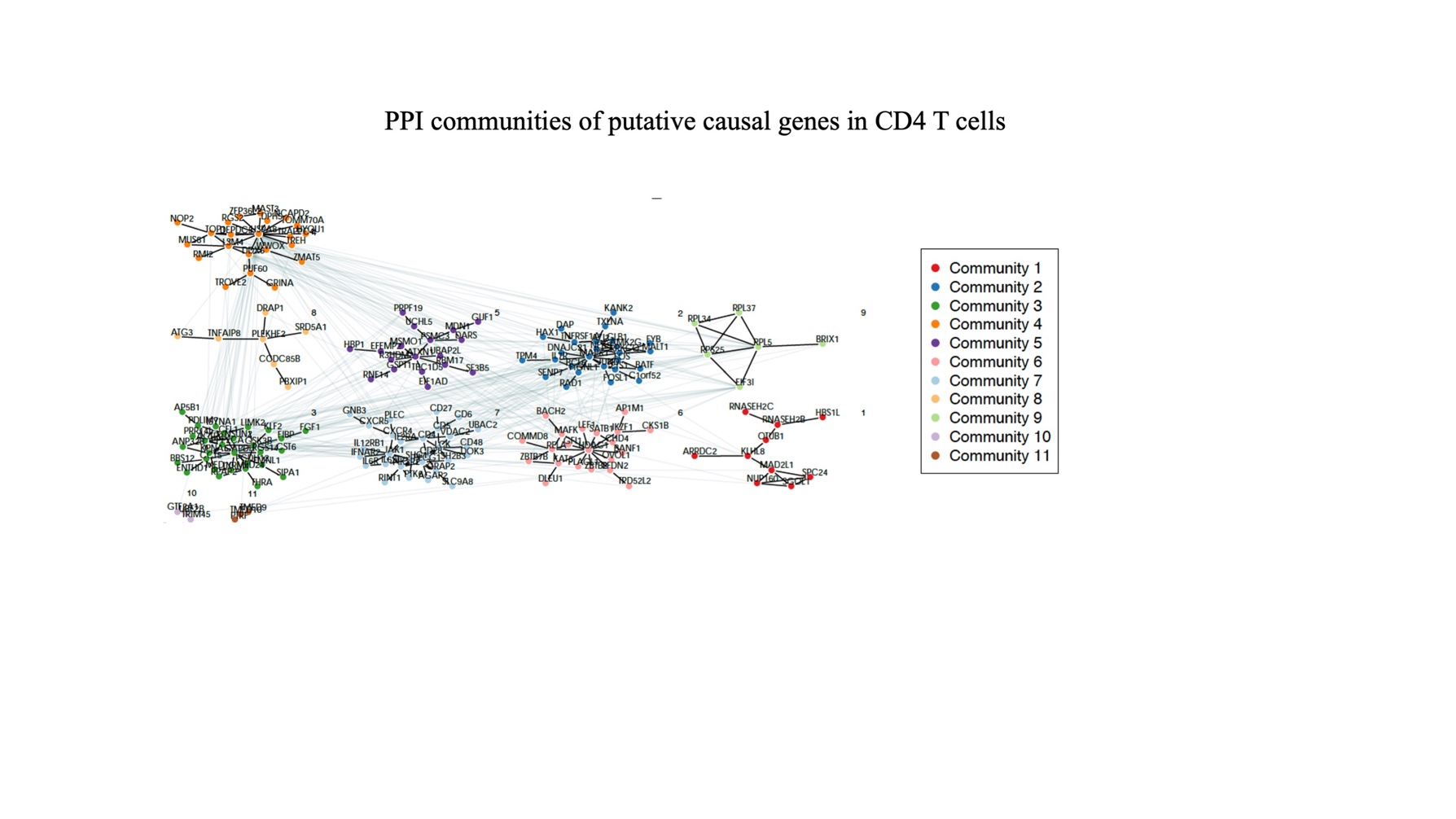


**Figure S12:** Protein-protein interaction communities of putative causal genes in B cells.


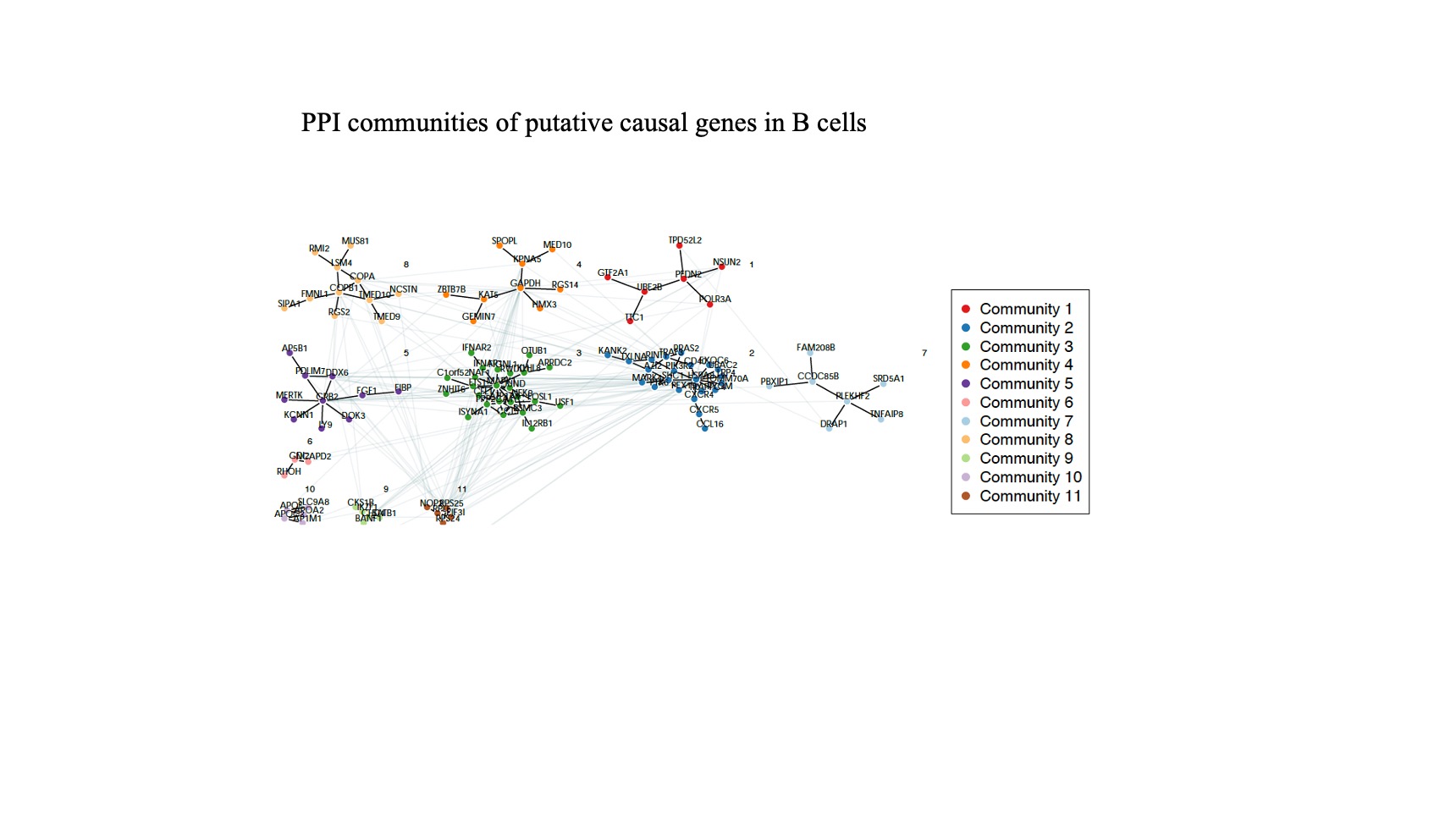


**Figure S13:** Protein-protein interaction communities of putative causal genes shared in CD4 T and B cells.


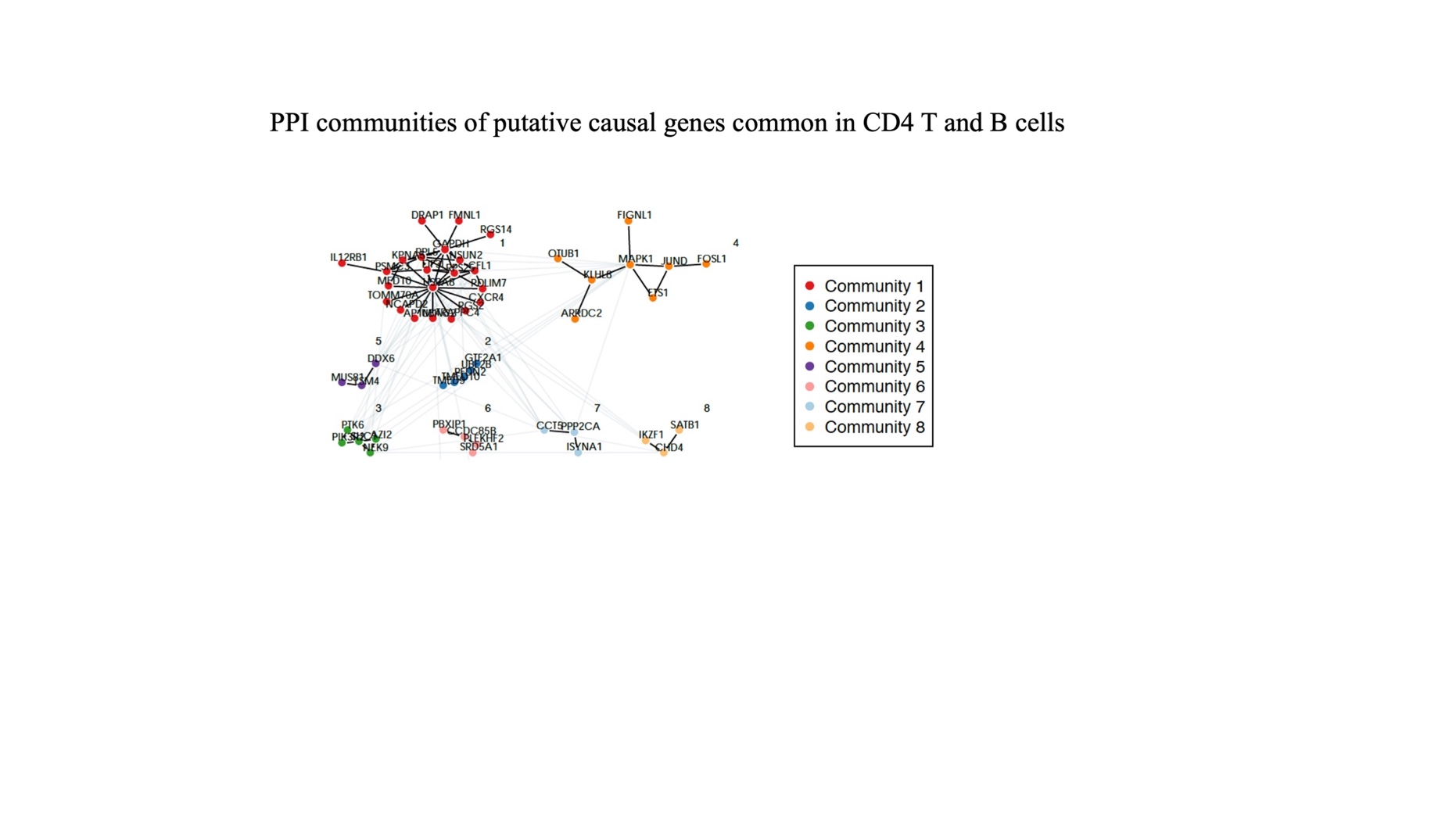


**Figure S14:** Protein-protein interaction communities of putative causal genes unique in CD4 T cells.

**
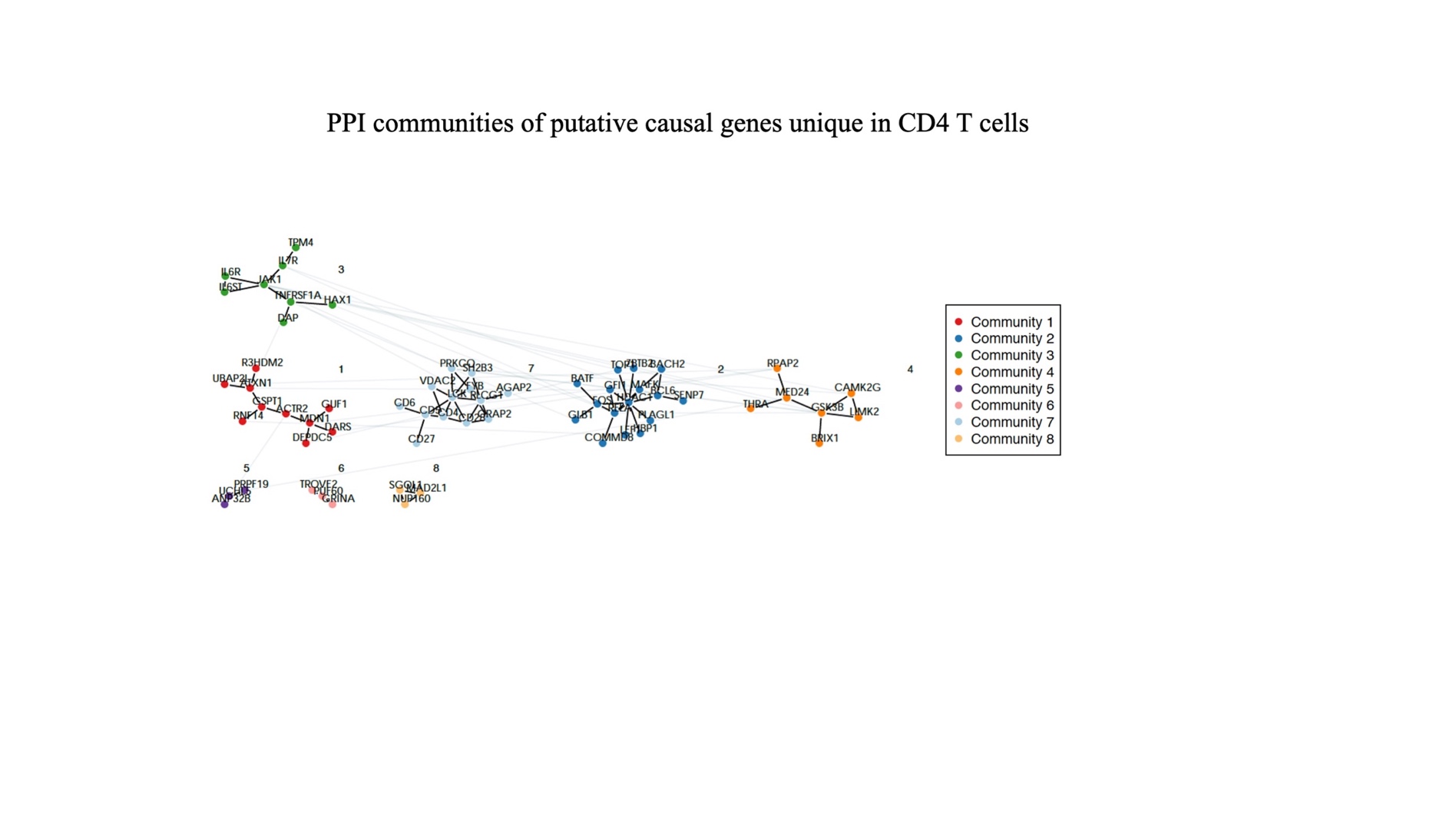
**

**Figure S15:** Protein-protein interaction communities of putative causal genes unique in B cells.


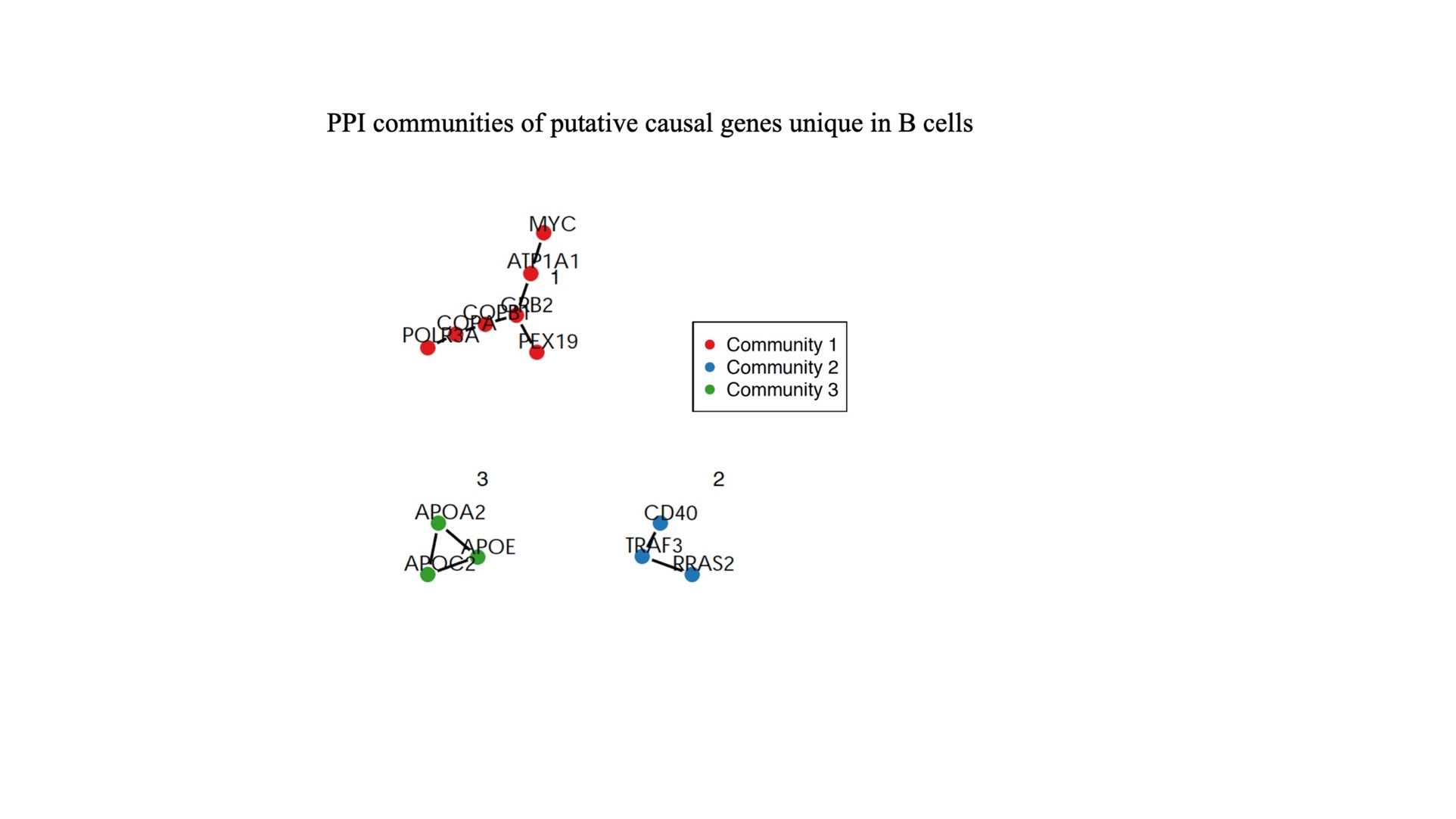


**Figure S16:** Enrichment of CD4 T cell putative causal genes in GTRD database. Each dot represents one transcription factor. The Y axis displays -log10 of false discovery rate (FDR). The dashed red line indicates the threshold of 1% FDR. The enrichment for TEAD2 predicted target genes is labeled.

**Figure S17:** Enrichment of B cell putative causal genes in GTRD database. Each dot represents one transcription factor. The Y axis displays -log10 of false discovery rate (FDR). The dashed red line indicates the threshold of 1% FDR. The enrichment for TEAD2 predicted target genes is labeled.


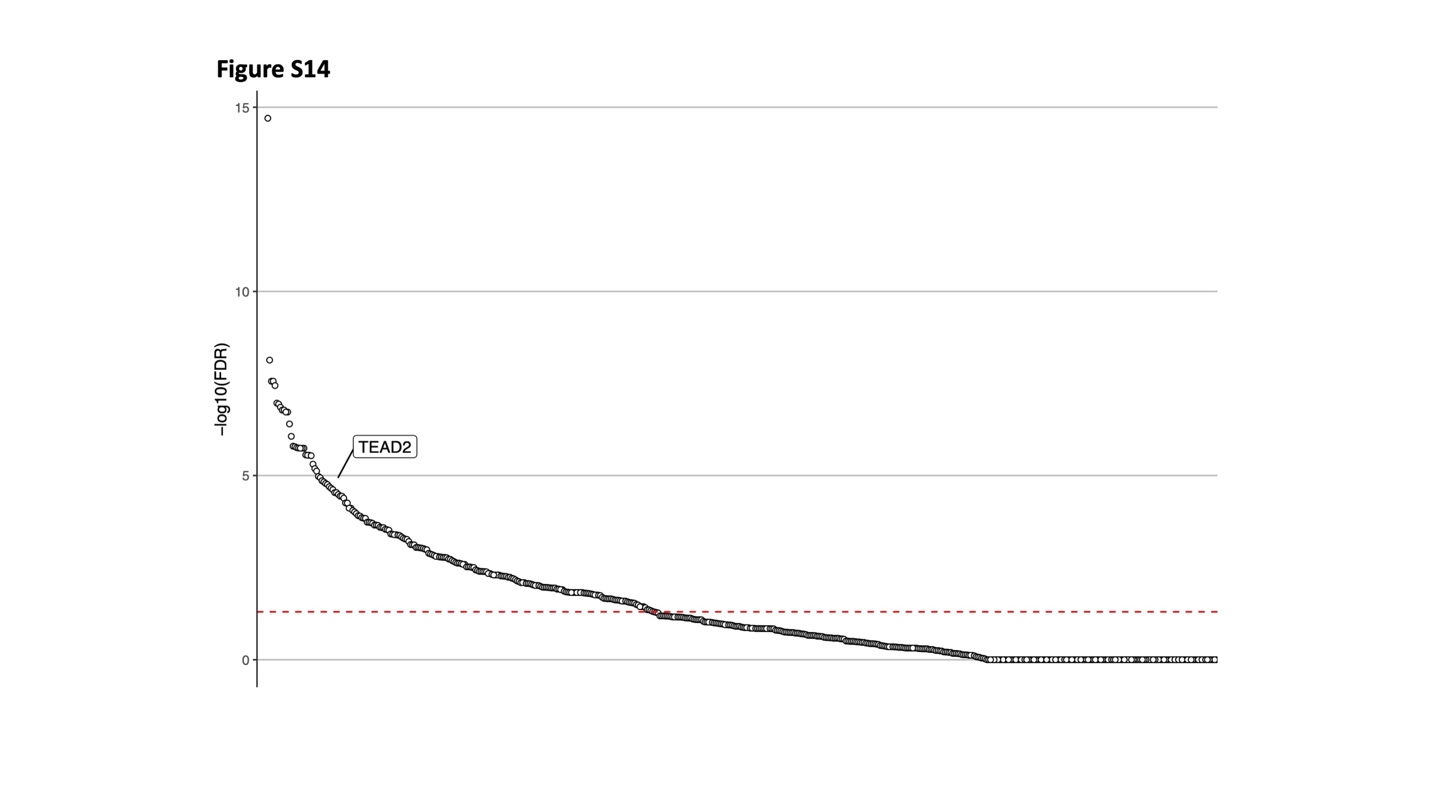


**Figure S18:** Enrichment of shared between CD4 T and B cells putative causal genes in GTRD database. Each dot represents one transcription factor. The Y axis displays -log10 of false discovery rate (FDR). The dashed red line indicates the threshold of 1% FDR. The enrichment for TEAD2 predicted target genes is labeled.


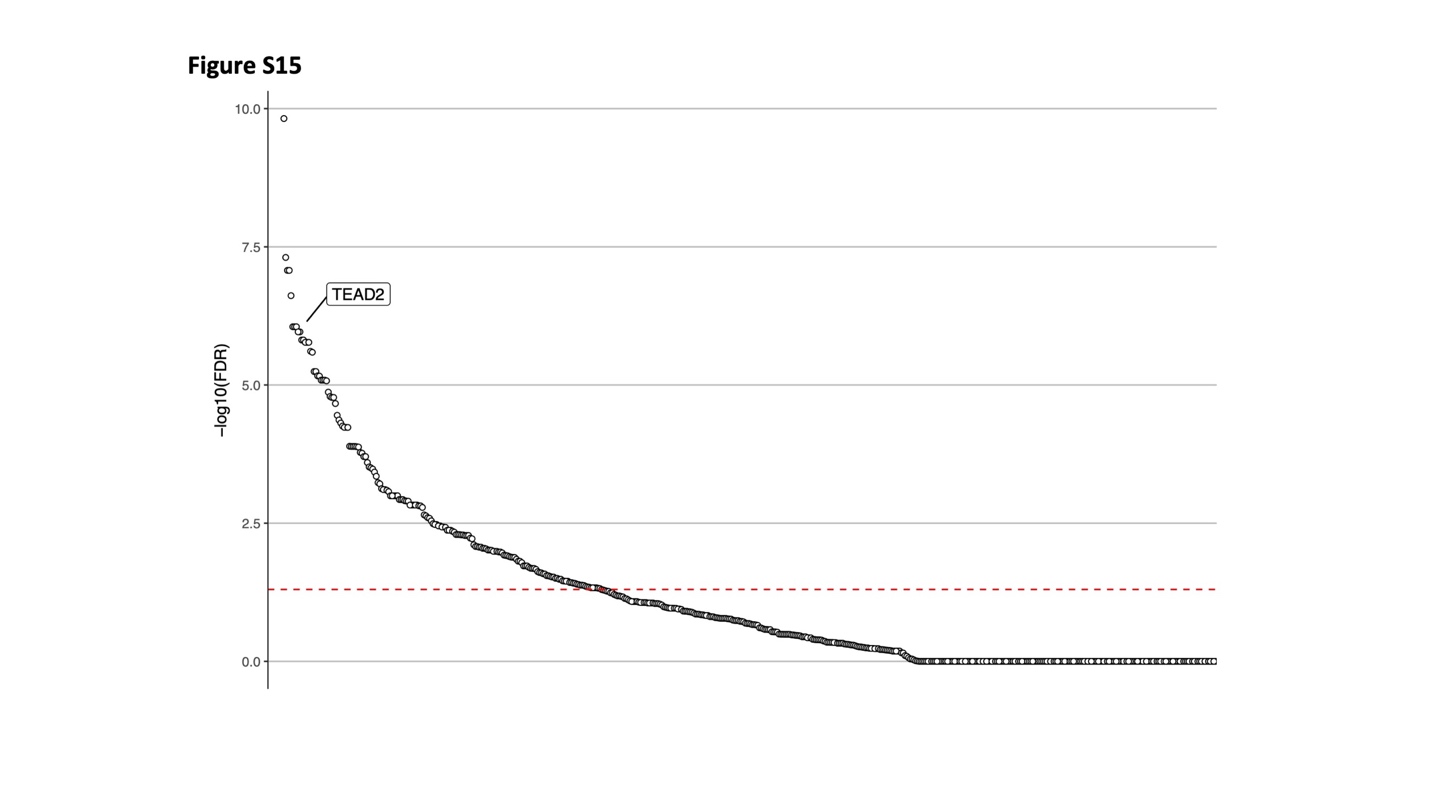


**Figure S19:** Change of gene expression of CD4 T cell putative causal genes in knock-down (KD) and over-expression (OE) models in cancer cell lines. Eight cancer cell lines are displayed: A375, A549, HA1E, HEPG2, HT29, MCF7, PC3, and VCAP. Putative causal genes are represented with lines connecting the ranked KD gene expression data (left column) with the ranked OE gene expression data (right column). Genes that are in the extreme 10% in opposite directions are indicated with green solid lines or red solid lines if these are also a predicted gene target for TEAD2. The light grey lines display all over putative causal genes.


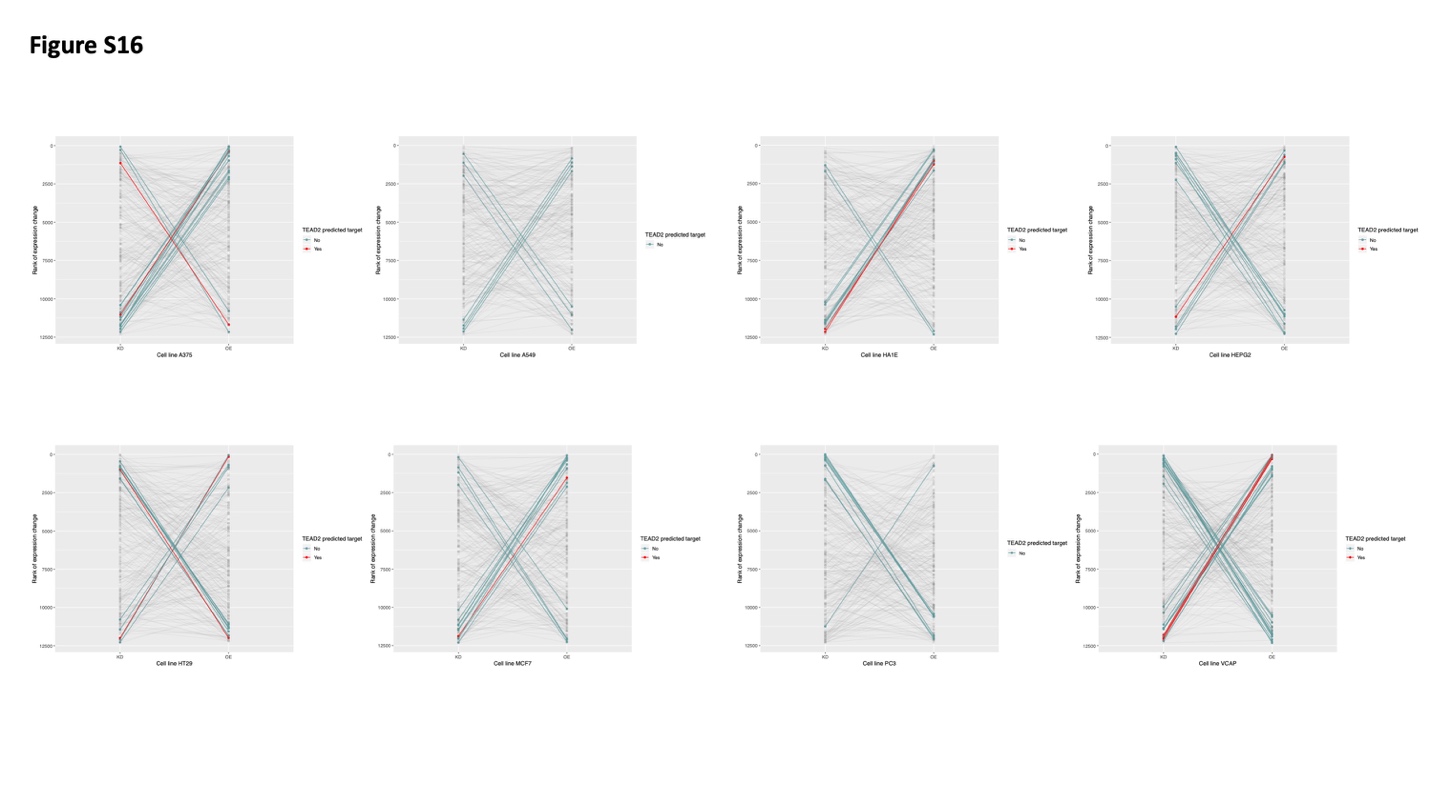


**Figure S20:** Change of gene expression of B cell putative causal genes in knock-down (KD) and over-expression (OE) models in cancer cell lines. Eight cancer cell lines are displayed: A375, A549, HA1E, HEPG2, HT29, MCF7, PC3, and VCAP. Putative causal genes are represented with lines connecting the ranked KD gene expression data (left column) with the ranked OE gene expression data (right column). Genes that are in the extreme 10% in opposite directions are indicated with green solid lines or red solid lines if these are also a predicted gene target for TEAD2. The light grey lines display all over putative causal genes.


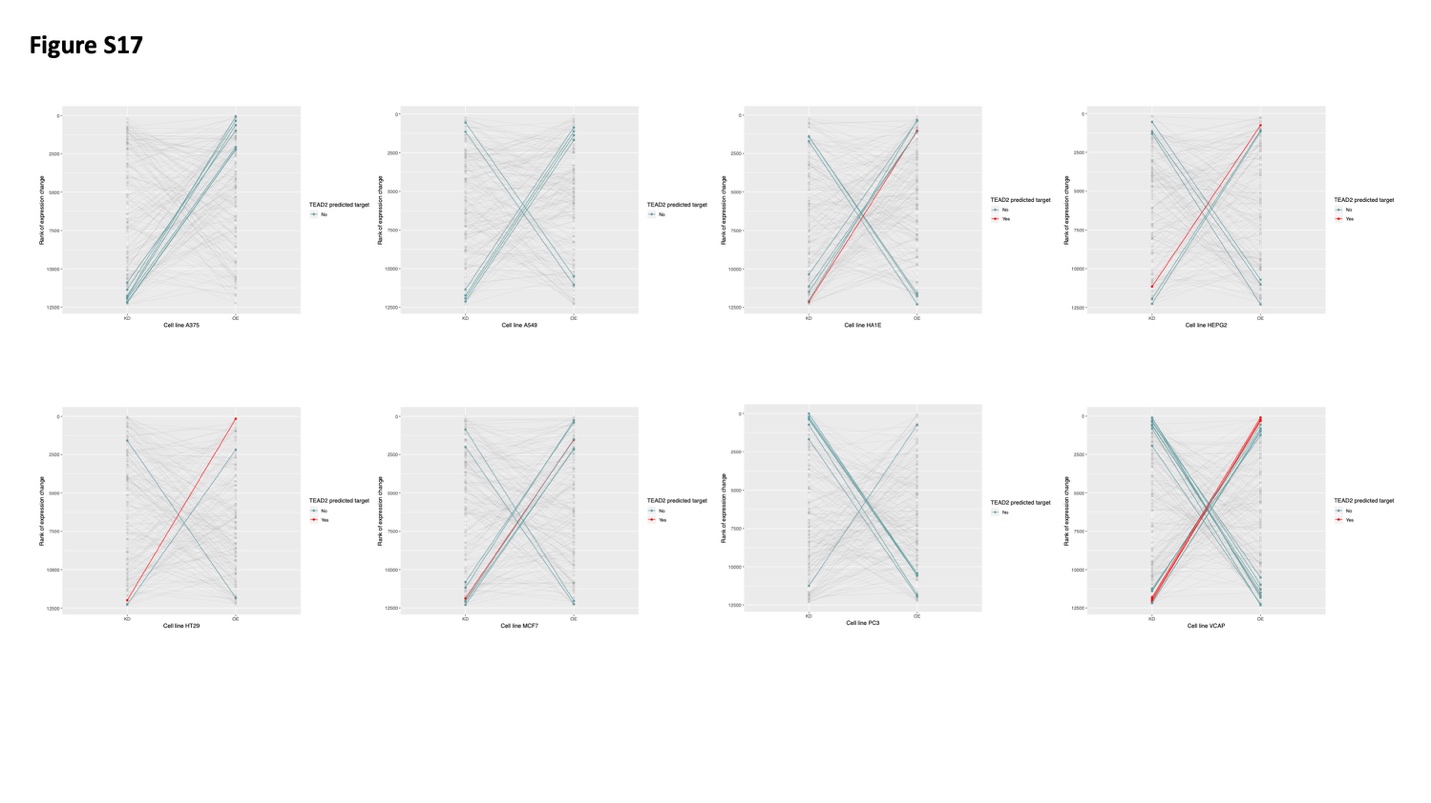

Supplement: Supplementary file 2 — Additional file 2: Supplementary Figures. Figure S1. Correlation in ATAC-seq profiles across hematopoietic cell types. Figure S2. LDSC enrichments for MS GWAS in cell-type specific ATAC-seq peaks mature hematopoietic cell type. Figure S3. Enrichments of GWAS results from 10 neuropsychiatric or autoimmune conditions in OCRs across various hematopoietic cell types. Figure S4. LDSC results for MS GWAS in ATAC-seq peaks from treated MS patients. Figure S5. LDSC results for MS GWAS in histone ChIP-seq. Figure S6. LDSC results for MS GWAS in chromHMM partitions. Figure S7. Distribution of credible set variants. Figure S8. Number of GWAS loci with ATAC-seq peak overlapping fine-mapped SNP. Figure S9. Colocalization of MS GWAS loci with DICE CD4 T and B cell eQTLs. Figure S10. Colocalization enrichment of MS GWAS loci with DICE CD4 T and B cell eQTLs. Figure S11. Protein-protein interaction communities of putative causal genes in CD4 T cells. Figure S12. Protein-protein interaction communities of putative causal genes in B cells. Figure S13. Protein-protein interaction communities of putative causal genes shared in CD4 T and B cells. Figure S14. Protein-protein interaction communities of putative causal genes unique in CD4 T cells. Figure S15. Protein-protein interaction communities of putative causal genes unique in B cells. Figure S16. Enrichment of CD4 T cell putative causal genes in GTRD database. Figure S17. Enrichment of B cell putative causal genes in GTRD database. Figure S18. Enrichment of shared between CD4 T and B cells putative causal genes in GTRD database. Figure S19. Change of gene expression of CD4 T cell putative causal genes in knock-down (KD) and over-expression (OE) models in cancer cell lines. Figure S20. Change of gene expression of B cell putative causal genes in knock-down (KD) and over-expression (OE) models in cancer cell lines. [file 13059_2022_2694_MOESM2_ESM.docx]
