# Supplementary material for: Dissection of multiple sclerosis genetics identifies B and CD4+ T cells as driver cell subsets
Source: Genome Biol. 2022 Jun 7;23:127. doi: 10.1186/s13059-022-02694-y (PMC9175345; doi:10.1186/s13059-022-02694-y)
Supplement: Supplementary file 3 — Additional file 3. Review history. [file 13059_2022_2694_MOESM3_ESM.docx]

Review History

**First round of review**

**Reviewer 1**

**Are you able to assess all statistics in the manuscript, including the appropriateness of statistical tests used?** Yes, and I have assessed the statistics in my report.

**Comments to author:**

In this article, the authors have used chromatin accessibility data (bulk ATAC-seq) across sorted hematopoietic cells to identify enrichments of MS genetic signals. The authors found that CD4 T and B cells were independently enriched for MS genetics. While some of the findings are interesting, the study lacks novelty and does not dive deep into exploring the relationships of causal variants.

Major comments

1. The whole concept of treating CD4 T cells and B cells as homogenous population is flawed and goes against the concept that the authors themselves raised "... whether different mechanisms are present in distinct immune cell populations." By just sorting CD4 T and B-cells, and performing bulk ATAC-seq, they are ignoring the heterogeneity in these populations. Additional data using single-cell ATAC-seq in broad hematopoietic cells will tremendously help this project. The authors could choose to generate their own data or integrate and re-analyze published snATAC-seq datasets.
2. The authors missed a critical opportunity to address partitioned heritability in these cell-types. Added analysis in this direction should be explored.
3. The amount of analysis is too weak and too preliminary, sophisticated analysis of fine-mapping can and should be done to further explore the relationships of causal variants in the LD region and integrate Hi-C and other data modalities.

**Reviewer 2**

**Are you able to assess all statistics in the manuscript, including the appropriateness of statistical tests used?** Yes, and I have assessed the statistics in my report.

**Comments to author:**

Guo et al. used chromatin accessibility (ATAC-seq) data to demonstrate the enrichment of Multiple Sclerosis (MS) variants in CD4 T cell and B cells, specifically Th17 and memory B cells. They have also employed fine mapping and reference PCHiC interaction data to identify the putative causal genes linked with MS variants. The authors have primarily employed stratified LD score regression (LDSC) technique to show the enrichment of MS variants in the open chromatin regions (OCR) of respective immune cells. However, I find some of the work and conclusions a bit repetitive (see my comments below). The authors should prepare a major revision of this manuscript by focusing more on the Th17 and memory B cells and show multiple evidences (comments below) of the enrichment of MS variants and putative causal genes in those immune cell subsets.
Major Comments
1. MS has long been declared as a disease related to CD4 T cell and B cells (see the reviews in PMID: 29358315, 32457742). Thus, the results in Figs. 2 and 3 are not informative in my opinion. Authors can retain the Fig. 1 to introduce the given dataset and LDSC framework, and focus more on the Th17 and memory B subsets (Fig. 4).
2. The results using LDSC to highlight the importance of Th17 and memory B cells (Figs. 4 and 5) are a bit repetitive. The joint model to highlight the contribution of a specific cell type is credible, and Figs. 4C and 5C are sufficient to highlight the respective cell types, without the need for Figs. 4D and 5D.
3. The manuscript needs to show the enrichment of MS GWAS variants in the eQTLs from Th17 and memory B cells compared to other CD4 T and B cell subtypes, to support the proposed claim. They can use eQTLs from eQTL Catalogue, DICE or ImmuNexUT databases.
4. Authors should check the colocalization between eQTLs and MS GWAS variants to show whether the Th17 and memory B cells exhibit higher number of colocalized variants, which are in the OCRs and having PCHiC contacts with their putative causal genes.
5. Authors have already employed fine mapping of MS variants. Are those fine mapped SNPs are enriched in OCRs from Th17 and memory B cells, compared to the other memory cells? And do those fine mapped MS variants show enrichment in terms of their overlap or LD with eQTLs from Th17 and memory B cells, compared to the other CD4 and B cell subtypes?
6. Using the reference PCHiC interactions, authors need to show the enrichment of PCHiC interactions involving the fine mapped variants for Th17 and memory B cells, compared to the other cell types, to justify that the proposed cell types are indeed enriched for the MS variants and their PCHiC connections with the putative causal genes.
Minor comments
1. Fig. S4A, S4B, S5A, S5B: the figure shows CD4 T cell but the corresponding text (section: MS GWAS signals in B and CD4 T cells driven by active enhancer and promoter regions) mentions Th17 cells - clarify.

**Authors Response**

**Point-by-point responses to the reviewers’ comments:**

Reviewer #1: In this article, the authors have used chromatin accessibility data (bulk ATAC-seq) across sorted hematopoietic cells to identify enrichments of MS genetic signals. The authors found that CD4 T and B cells were independently enriched for MS genetics. While some of the findings are interesting, the study lacks novelty and does not dive deep into exploring the relationships of causal variants.

Major comments

1. The whole concept of treating CD4 T cells and B cells as homogenous population is flawed and goes against the concept that the authors themselves raised "... whether different mechanisms are present in distinct immune cell populations." By just sorting CD4 T and B-cells, and performing bulk ATAC-seq, they are ignoring the heterogeneity in these populations. Additional data using single-cell ATAC-seq in broad hematopoietic cells will tremendously help this project. The authors could choose to generate their own data or integrate and re-analyze published snATAC-seq datasets.

**We thank the reviewer for this comment. Indeed, CD4 T and B cells are not homogenous and single cell studies are paving the road to capture their level of heterogeneity. Unfortunately, current published single cell ATAC-seq studies lack the required depth and sample size to perform meaningful analyses. Available single cell ATAC-seq technologies trade off OCR capture and sequencing depth for number of quantified cells and low cost, resulting in heavily sparse data with shallow sequencing depth. Furthermore, current single cell ATAC-seq datasets from human immune cells are not large enough to provide more granular insights beyond the sorted high-quality and sequencing depth cell populations we analyzed. Additionally, existing methods for integrating single-cell ATAC data with GWAS results are relatively nascent and most methods still require creating pseudo-bulk OCRs from the single cell ATAC-seq, thus limiting the utility of using scATAC-seq. Despite these limitations, we attempted to apply gchromVAR, a method to integrate fine-mapping data with scATAC-seq data (Lareau et al., Nature Genetics. 2020). We applied gchromVAR to single cell ATAC-seq data from 4146 PBMCs from Satpathy et al. Nature Biotechnology. 2019. We performed fine-mapping using PICS and then projected the fine-mapping results on normalized single cell ATAC-seq peak matrices using gchromVAR. Application of gchromVAR did not provide any clear patterns of cell-type enrichment for MS fine-mapping data (Figure R1). In this plot, each point is a single cell and is projected on UMAP 1 and 2. The color of each point reflects the z-score of the enrichment of MS GWAS in peaks from that cell. We believe that this lack of single cell enrichment is due to a combination of sparse single cell data and imperfect statistical fine-mapping.**

**As this will certainly be an area of great importance and future exploration, we have added additional text to our Discussion (lines 447-450). Finally, we agree with the reviewer that single cell ATAC-seq data generation will be helpful, but this need to be performed at large scale, utilizing sorted populations (e.g., CD4 T cells) to allow for the discovery of heterogeneous rare cells, high sequencing depth, and most probably bulk-level data generation of sorted newly discovered cells to allow for in-depth integration of MS genetics. We believe our paper will be an extremely valuable resource and the starting point for these next level set of projects.**

2. The authors missed a critical opportunity to address partitioned heritability in these cell-types. Added analysis in this direction should be explored.

**We are unsure what the reviewer is referring to with this comment, as our manuscript applies partitioned heritability from LDSC throughout.**

3. The amount of analysis is too weak and too preliminary, sophisticated analysis of fine-mapping can and should be done to further explore the relationships of causal variants in the LD region and integrate Hi-C and other data modalities.

**Thank you for this important comment, which was also partially raised by Reviewer #2. To address this, we have performed various enrichment analyses of ATAC-seq, PCHiC, and eQTL data with our statistical fine-mapping data. We also note that Supplemental Table S24 integrates these extensive orthogonal datasets with the MS GWAS data and statistical fine-mapping data. This table allows readers to easily browse for variants of interest and understand their potential epigenetic, transcriptomic, and 3D genome consequences.**

Reviewer #2: ===

Guo et al. used chromatin accessibility (ATAC-seq) data to demonstrate the enrichment of Multiple Sclerosis (MS) variants in CD4 T cell and B cells, specifically Th17 and memory B cells. They have also employed fine mapping and reference PCHiC interaction data to identify the putative causal genes linked with MS variants. The authors have primarily employed stratified LD score regression (LDSC) technique to show the enrichment of MS variants in the open chromatin regions (OCR) of respective immune cells. However, I find some of the work and conclusions a bit repetitive (see my comments below). The authors should prepare a major revision of this manuscript by focusing more on the Th17 and memory B cells and show multiple evidences (comments below) of the enrichment of MS variants and putative causal genes in those immune cell subsets.

Major Comments

1.     MS has long been declared as a disease related to CD4 T cell and B cells (see the reviews in PMID: 29358315, 32457742). Thus, the results in Figs. 2 and 3 are not informative in my opinion. Authors can retain the Fig. 1 to introduce the given dataset and LDSC framework, and focus more on the Th17 and memory B subsets (Fig. 4).

**While we agree that the role of both CD4 T and B cells in MS is now considered established knowledge, this was an outcome of decades of work and debate among the scientific community (the provided reviews capture in part that trajectory). In this paper, we provide casual explanation to their disease importance by integrating MS genetics with cell-specific open chromatin data. The fact that the agnostic integration of orthogonal data published by different groups points to the same answer that was formed over hundreds of studies is exciting. Furthermore, these results show that the CD4 and B cell enrichments are conditionally independent of OCRs in other cell types, providing evidence of common and cell-type specific causal associations with MS genetics. However, we agree that the results can be further streamlined and we have moved previous Figure 3 to the Supplement Figure S3, trimming the respective text.**

2.     The results using LDSC to highlight the importance of Th17 and memory B cells (Figs. 4 and 5) are a bit repetitive. The joint model to highlight the contribution of a specific cell type is credible, and Figs. 4C and 5C are sufficient to highlight the respective cell types, without the need for Figs. 4D and 5D.

**Thank you for this suggestion to streamline our manuscript. We have removed previous Figures 4D and 5D, along with their associated text.**

3.     The manuscript needs to show the enrichment of MS GWAS variants in the eQTLs from Th17 and memory B cells compared to other CD4 T and B cell subtypes, to support the proposed claim. They can use eQTLs from eQTL Catalogue, DICE or ImmuNexUT databases.

**We thank for the reviewer for this comment, which adds another layer of evidence towards causal association between genes and GWAS loci. Although there is added value in colocalizing a GWAS locus with an eQTL, we caution that lack of colocalization does not translate to lack of causality. Moreover, there are several limitations in utilizing eQTLs to further refine and compare subsets of CD4 T and B cells. First, there is a strong assumption that most, if not all, disease associated variants mediate their effect via direct regulation of gene expression. Second, that the regulated genes are expressed in the assayed cells, regardless of the sample origin (e.g., healthy individual vs. subjects with the diseases). Third, that the available studies are large enough to identify such effects. Finally, that the eQTL studies have a comprehensive enough list of cell subtypes to allow for informative comparisons.**

**Of the currently available human immune cell eQTL data sets, DICE and ImmuNexUT have studied subsets of CD4 T cell or both CD4 T and B cells (DICE includes only naïve B cells). Of the two, DICE has a sample size of 91 and ImmuNexUT (up to) 416 subjects. DICE included healthy controls of European ancestry and ImmuNexUT a mixture of control and subjects with various autoimmune/inflammatory non-MS diseases of Asian ancestry. In response to the reviewer’s comment, we applied Mediated Expression Score Regression (MESC; PMID: 32424349). MESC utilizes eQTL data and GWAS summary statistics to identify cells/tissues whose gene expression is mediated by the phenotype associated genetic variation. MESC requires large sample size to lead to interpretable results (> 500; “We observed that MESC produced unbiased or nearly unbiased estimates of ℎ2/ℎ2 across all simulated genetic architectures with expression panel sample size greater than 500 when using the best-performing method to estimate expression scores, LASSO with REML correction”) and of the two available eQTL data sets ImmuNexUT has the largest sample size. We applied MESC for MS GWAS summary statistics and the CD4 T and B cell subsets of ImmuNexUT in three ways: i) using all the cis-eGenes, ii) creating per cell subset gene sets of eGenes unique compared to the other subtypes of the same population (e.g. Th17 vs. the rest of CD4 T cells), and iii) creating per cell subset gene sets of eGenes unique compared to CD4 T and B cell subtypes (e.g. Th17 vs. other CD4 T cells and all B cells). We observed unstable results, especially in CD4 T cells, possibly due to a combination of the sample size and the vastly different ancestry of the ImmuNexUT subjects (see Table R1 below).**

**Table R1. Estimated proportion of heritability mediated by the cis-genetic component of assayed gene expression levels (ℎ2/ℎ2) with respective standard error (SE).**

**Cell	Population	h2med over total heritability	SE**

**Th17	CD4 T	-0.0115189	0.08140269**

**Fr_II_eTreg	CD4 T	0.08967084	0.07614119**

**Fr_I_nTreg	CD4 T	-0.0243424	0.08839989**

**Th2	CD4 T	0.04777865	0.07865432**

**Th1	CD4 T	0.04138503	0.07226733**

**Naive_CD4	CD4 T	-0.0943111	0.08527741**

**Tfh	CD4 T	-0.0221698	0.08957781**

**Mem_CD4	CD4 T	0.01118831	0.08013737**

**Fr_III_T	CD4 T	0.0028385	0.08126742**

**SM_B	B cells	0.18288068	0.0763352**

**USM_B	B cells	0.10658559	0.0915444**

**Naive_B	B cells	0.17986622	0.06862532**

**DN_B	B cells	0.14767879	0.08284797**

**MESC splits genes into 5 bins by magnitude of expression cis-heritability to estimate the GWAS enrichment. Across all cell types, CD4 T and B, only 4 bins (two in Naïve_B, one in SM_B, and one in USM_B) had an enrichment p-value < 0.05, with enrichment 0.009x to 0.04x i.e., almost non-existent. Both gene set analyses resulted in no enrichment at p-value <0.05 in any cell subtype. These results reflect the lack of utility of the current immune cell eQTL data sets for such analyses due to small sample size and different ancestry.**

**We believe that the currently available eQTL studies are not informative enough to answer this question. Additionally, we agree with other groups that eQTL studies is not the ideal mechanism to identify the causal relationship of genetics in common diseases, which are in their majority of regulatory nature. A recent preprint by Conally et al (https://www.medrxiv.org/content/10.1101/2021.06.08.21258515v2) elaborate in detail on this argument (e.g. from the abstract: “These results contradict the hypothesis that most complex trait-associated variants coincide with currently ascertained expression quantitative trait loci. The field must confront this deficit, and pursue this “missing regulation.” “). Although the reviewer’s comment is an interesting one, we do not believe that including unstable and underpowered results will benefit the reader.**

4.     Authors should check the colocalization between eQTLs and MS GWAS variants to show whether the Th17 and memory B cells exhibit higher number of colocalized variants, which are in the OCRs and **having PCHiC contacts with their putative causal genes.**

**We agree with the reviewer that this is an interesting analysis. Despite the above-mentioned limitations of utilizing eQTLs to address these questions, we performed colocalization analysis with coloc and both the DICE and ImmuNexUT set of results. ImmuNexUT did result in colocalized loci-eGenes but there was little overlap with the DICE eGenes. This is driven predominantly by the different ancestry (largely East Asian for ImmuNexUT; European for MS) and we decided not to include these results, although ImmuNexUT had the largest sample size and more granular cell subtypes. For the DICE results, we observed about a third of colocalized loci-eGenes to overlap with OCRs+loops. Examining enrichment of overlap including all tested loci-eGenes, we observed strong enrichment for naïve Tregs and Th17. We have included in the main text a paragraph summarizing these results (lines 328-338, along with Supplementary Figures S9-S10. We would like to add a cautionary note that colocalization can be affected by several factors, including the input list of regional variants and underlying haplotypic structure on both datasets. Thus, while colocalization can identify highly robust variant-gene associations but lack of localization does not translate to lack of true causal biological associations.**

5.     Authors have already employed fine mapping of MS variants. Are those fine mapped SNPs are enriched in OCRs from Th17 and memory B cells, compared to the other memory cells? And do those fine mapped MS variants show enrichment in terms of their overlap or LD with eQTLs from Th17 and memory B cells, compared to the other CD4 and B cell subtypes?

**We thank the reviewer for this suggestion. We have examined the enrichment of fine-mapped SNPs and found that memory B cell and Th17 CD4 T cell OCRs are in fact enriched in our credible set variants. Furthermore, these enrichments are maintained when examining OCRs present specifically in these cell types. These data reinforce our approach of integrating epigenetic datasets with statistical fine-mapping results for prioritization of potentially causal variants. We have added additional text (lines 302-310) and tables (Supplementary Tables S24-25).**

6.     Using the reference PCHiC interactions, authors need to show the enrichment of PCHiC interactions involving the fine mapped variants for Th17 and memory B cells, compared to the other cell types, to justify that the proposed cell types are indeed enriched for the MS variants and their PCHiC connections with the putative causal genes.

**We thank the reviewer for this suggestion. We have examined the enrichment of fine-mapped SNPs and found that among all cell types, PCHiC loops from naïve B cells and activated CD4 T cells have the strongest enrichments. These data provide additional support for our approach of integrating chromatin looping with statistical fine-mapping results for prioritization of potentially causal variants. We have added additional text (lines 320-326) and tables (Supplementary Tables 26)**

Minor comments

1.     Fig. S4A, S4B, S5A, S5B: the figure shows CD4 T cell but the corresponding text (section: MS GWAS signals in B and CD4 T cells driven by active enhancer and promoter regions) mentions Th17 cells - clarify.

**We have revised the text to clarify that these are in fact Th17 subset of CD4 T cells.**

**Second round of review**

**Reviewer 1**

The authors have sufficiently addressed several points from earlier revision, the work merits publication without further review

**Reviewer 2**

Most of my comments are addressed. Below find some minor issues:
Line 329: gene eQTLs -> eQTLs.
Line 333: eQTL eGene -> eGene. loci-eGenes -> SNP-gene
Line 329: "colocalize with these epigenetic features" it is not the colocalization with epigenetic features, it is the overlap of colocalized SNPs
- Line 625: "any eGene that had an FDR of 10% or less" -> any eGene with a significant eQTL association at 10% FDR
For coloc analysis, the posterior probability threshold for H4 (PP4) is lower than suggested by the tool. This has to be justified.

**Authors Response**

**Point-by-point responses to the reviewers’ comments:**

**We are pleased to hear that Reviewer #1 is satisfied with our revisions. We thank Reviewer #2 for his/her suggestions, which we have addressed below:**

•Line 329: gene eQTLs -> eQTLs. **We have made the requested change**

•Line 333: eQTL eGene -> eGene. loci-eGenes -> SNP-gene: **We have made the requested change.**

•Line 329: "colocalize with these epigenetic features" it is not the colocalization with epigenetic features, it is the overlap of colocalized SNPs. **We have revised this to read: “colocalize SNPs overlapping these epigenetic features.”**

•Line 625: "any eGene that had an FDR of 10% or less" -> any eGene with a significant eQTL association at 10% FDR: **We have made the requested change.**

•For coloc analysis, the posterior probability threshold for H4 (PP4) is lower than suggested by the tool. This has to be justified.

**There is a wide range of H4 (PP4) used in the literature. For example, Gay et al Genome Biology volume 21, Article number: 233 (2020) used a threshold of >=0.5: “… to identify loci with high posterior probabilities of colocalization between eQTLs and GWAS (PP4 > 0.5)…”. We utilized a value of 0.6 to increase the number of observations for our overlap analysis. We have added a sentence to our manuscript at line 338 regarding this choice: “We note that we used a less stringent threshold of a colocalization posterior probability of 0.6 for these enrichment analyses to increase the number of observations for examining OCR overlap.”**
